# Comparative plastome analyses and evolutionary relationships of 25 East Asian species within the medicinal plant genus *Scrophularia* (Scrophulariaceae)

**DOI:** 10.3389/fpls.2024.1439206

**Published:** 2024-09-03

**Authors:** Xia Wang, Lei Guo, Lulu Ding, Leopoldo Medina, Ruihong Wang, Pan Li

**Affiliations:** ^1^ Zhejiang Province Key Laboratory of Plant Secondary Metabolism and Regulation, College of Life Sciences and Medicine, Zhejiang Sci-Tech University, Hangzhou, China; ^2^ Real Jardín Botánico, Consejo Superior de Investigaciones Científicas (CSIC), Madrid, Spain; ^3^ Laboratory of Systematic and Evolutionary Botany and Biodiversity, College of Life Sciences, Zhejiang University, Hangzhou, China

**Keywords:** *Scrophularia*, medicinal plant, East Asia, chloroplast genome, comparative analysis, phylogenomics

## Abstract

**Backgroud:**

*Scrophularia* L., a genus of the Scrophulariaceae, is a group of important medicinal plants used for eliminating heat and detoxifying. East Asia has an abundance of potentially medicinal *Scrophularia* species, and it serves as a secondary diversity center of the genus. However, the genomic resources available for germplasm identification and pharmaceutical exploration of East Asian *Scrophularia* are insufficient, hindering its commercial and industrial development. Additionally, the interspecific relationships of most East Asian *Scrophularia* species remain unclear.

**Methods:**

In this study, we sequenced the leaves of 25 East Asian species of the genus *Scrophularia*, assembled and annotated the complete chloroplast genomes, and subsequently performed comparative and phylogenetic analyses on these genomes.

**Results and discussion:**

The conserved plastome length of these 25 species ranged from 151,582 bp to 153,239 bp, containing a total of 132 coding genes, including 18 duplicated genes and 114 unique genes. Through genome alignment of these 25 species, 38-53 repeated sequences and 7 shared SSRs were identified, along with regions with high nucleotide polymorphism (Pi), which could potentially serve as molecular markers for species identification. The genome structure, gene content, and arrangement showed conservation, while variations were observed in the IR boundary regions and IGS. Phylogenetic inferences based on whole plastomes or on coding sequences (CDS) only yielded congruent results. We categorized the 25 East Asian *Scrophularia* species into six distinct clades and further explored their interspecies relationships using morphological characteristics, such as flower color, the relative position of stamens and corolla, and plant height. This could lay a genetic basis for future resource development of *Scrophularia* in East Asia.

## 1 Introduction

*Scrophularia* L., a species-rich and complicated genus of Scrophulariaceae, comprises nearly 300 species across the Northern temperate zone (Hong et al., 1998; Wang, 2015). Southern Europe and the Mediterranean are the main center of *Scrophularia* diversity (Hong, 1983), while East Asia is the secondary center with a total of 42 documented species, 36 of which are recorded in China (Li et al., 1999; Wang, 2015). Many species of this genus possess high therapeutic properties and are extensively employed as herbal medications to treat fever, constipation, rheumatism, and inflammatory affections (Huang, 2018; Lee et al., 2021; Cui, 2023). It is notable that *Scrophularia* plants possess bioactive iridoids, such as harpagide and harpagoside, which are found in most species of this genus (Li et al., 1999; de Santos Galíndez et al., 2002; Pasdaran and Hamedi, 2017). *Scrophularia ningpoensis* Hemsl., which has a wider distribution in China and was officially listed in the Chinese Pharmacopoeia as the sole medicinal source of Scrophulariae Radix (SR, also called “Xuan Shen”), has been used over two thousand years (Chinese Pharmacopoeia Commission, 2020; Lee et al., 2021). While the remaining species in China tend to exhibit distinct regional characteristics (**Supplementary Table 1**) and are usually utilized as local folk remedies or ethnic medicines, such as *S. spicata* Franch.*, S. fargesii* Franch., *S. henryi* Hemsl. and *S. moellendorffii* Maxim (Li et al., 1999). *S. incisa* Weinm. is a traditional Mongolian medicine, and its entire plant is used for treating measles and rash diseases (Editorial Board of the Chinese Materia Medica, 2003). *S. dentata* Royle ex Benth. is employed as an ethnic medicine known as “Alpine Tibetan herb” for the treatment of exanthema and fever (Zhang et al., 2013; Ni et al., 2016). *S. buergeriana* Miq., *S. kakudensis* Franch. and *S. yoshimurae* T. Yamaz., as the common substitution and adulterants of SR, have been employed in Korea and in Taiwan Province for over 20 years (Sagare et al., 2001; Nam et al., 2020; Manivannan et al., 2021; Guo et al., 2023b). However, due to restricted wild distribution and indiscriminate harvesting and exploitation, wild strains of *S. ningpoensis* are facing a diminishing genetic diversity (Wang and Wang, 2007; Zhao, 2008; Chen, 2014). Furthermore, *S. ningpoensis* is well-known as one of the “Zhe Ba Wei” (eight traditional Chinese medicines from Zhejiang Province), and it is grown in many other provinces across China by introduction breeding (Chen, 2011). Due to the decreasing cultivation area of authentic *S.ningpoensis* in Zhejiang (He et al., 2020), extensive cultivation in other provinces has intensified market competition (Zhang et al., 2022a). The intensification of market competition has led to an increase in artificial cultivation. However, owing to intensive artificial selection (Chen, 2011) and the unsustainable practices adopted by farmers, like long-term asexual reproduction, the genetic diversity of cultivated varieties and the quality of medicinal materials continue to decline (Yang, 2011). Therefore, it is crucial to clarify the species relationship of *Scrophularia* in East Asia and develop its medicinal resources reasonably. Efficient universal molecular markers are also essential to promote contemporary breeding projects in order to explore and conserve the germplasm of this medicinally and economically significant genus.

Despite the considerable medicinal worth of *Scrophularia* rhizomes, differentiating among species presents a challenge because of their analogous therapeutic properties and physical characteristics (Guo et al., 2023b). To understand species’ genetic background, since the 1990s, a multitude of approaches have been applied to explore the origin, genetic diversity and evolutionary relationships of *Scrophularia* (Ortega Olivencia and Devesa Alcaraz, 1993), including pollination system (Navarro-Pérez et al., 2013), plastid DNA datasets (e.g. *trnL*-*trnF*, *psbA*-*trnH*, *trnQ*-*rps16* and *trnS*-*trnG*), and nuclear ribosomal DNA sequences (nuclear ITS) (Attar et al., 2011; Scheunert and Heubl, 2011; Navarro-Pérez et al., 2013; Scheunert and Heubl, 2014; 2017). Among these efforts, the most extensive sampling of phylogenetic relationships within the *Scrophularia* genus to date has been constructed using sequences from the nuclear ITS region and two plastid DNA regions, encompassing 147 species worldwide, but only 13 East Asian species included (Scheunert and Heubl, 2017). In addition, several subclades and infrageneric relationships had weak support, especially within the East-Asian lineage of *S*. sect. *Scrophularia*. Considering this, further investigation of East Asian *Scrophularia* using more comprehensive genomic information, including the plastome, would be of great interest and significance for advancing our understanding in this field.

East Asia is a natural plant floristic region and one of the most diverse and complex regions in terms of plant biodiversity worldwide (Boufford and Ōba, 1998; Li et al., 2015). Once a vital ice age refuge, it’s now seen as a hub for angiosperm diversification and possesses one of the world’s oldest and most complete series of plant diversity evolution (Li, 2008). East Asiatic Floristic Kingdom reflects the evolutionary history and interrelationships of species, revealing the impact of environmental and ecological interactions (Deng, 2015), from the uplift of Qinghai-Tibetan Plateau (An and Harrison, 2000; Liu et al., 2006) to monsoonal climates (Azani et al., 2019). Therefore, East Asia is a critical region for the origin and evolution of angiosperms worldwide, as well as an important area and natural laboratory for studying distribution processes. Most of China is part of the East Asiatic Floristic Kingdom (Chen et al., 2022b), a young biome from the Early Miocene (Tang and Li, 1996; Milne and Abbott, 2002; Yang et al., 2023), rich in both relict plants (Milne and Abbott, 2002) and young species. Investigating the inter-species relationships within East Asian *Scrophularia* could provide theoretical support for applications such as molecular plant breeding and the analysis of medicinal components, while also offering a useful perspective on the evolution of East Asian *Scrophularia* species.

Genomic data provide us with a convenient tool to explore inter-specific phylogeny and mechanisms of intra-specific differentiation (Lin, 2020; Chen et al., 2021; Zhang et al., 2022b). Despite some limitations, such as challenges in addressing incomplete lineage sorting (ILS) and hybridization, plastids with their conserved structure and low recombination are still valuable for sequencing and phylogenetic analysis in angiosperms (Daniell et al., 2016; Feng et al., 2022; Xiang et al., 2024). Overall, plastome sequences have been commonly utilized to build phylogenies for plants (Raubeson et al., 2007; Gao et al., 2010; Lin, 2020).

In this study, we compared and characterized the complete plastomes of 25 species of East Asian *Scrophularia*, with respect to checking the contraction and expansion of the IR regions, identifying rapidly evolving regions of plastid DNA (i.e. SSRs and differentiation hotspots such as repeat sequence) and calculating the protein-coding genes selective pressure. Another goal was to build a strongly supported phylogenetic trees of 44 plastomes, elucidating their evolutionary relationships. These research efforts are expected to be helpful for future research on medicinal resource development, cultivation and breeding, species identification, systematics and conservation of *Scrophularia*.

## 2 Material and methods

### 2.1 Plant sampling, DNA extraction, sequencing, assembly and annotation

Leaves of 25 East Asian *Scrophularia* species were collected for sequencing (**Supplementary Table 2**). Voucher specimens were preserved in the Zhejiang University Herbarium (HZU). The CTAB method with modifications was employed to extract DNA from silica gel-dried leaves (Zhou et al., 2021). The lysis reagent Plant DNAzol (Invitrogen Corp. CTAB lysis solution) was used to extract the total genomic DNA from the leaf material of *Scrophularia* species. The MGIEasy Universal DNA Library Prep Set (96 RXN, Item No.: 1000006986) was used for the preparation of total libraries, which were then sequenced on the DNBSeq platform using the PE100 strategy at China National GenBank (CNGB) in Shenzhen, China. After obtaining the raw sequencing data, low-quality reads and adapters were filtered out using Trimmomatic v0.39 (Bolger et al., 2014). Through read mapping and gap-filling steps, GetOrganelle software was utilized iteratively *de novo* to assemble the complete plastome. Geneious software (Geneious Biologics 2023 (https://www.geneious.com/biopharma/, accessed on 10 May 2023)) was used to annotate the assembled plastomes and the annotations of *rps12* gene and *ycf1* gene was inspected with CPGview (Liu et al., 2023). Eventually, the complete plastomes of 25 East Asian *Scrophularia* species were all uploaded to NCBI Genbank database.

### 2.2 Repeat sequences, SSRs and codon usage bias analysis

We used the online tool REPuter (https://bibiserv.cebitec.uni-bielefeld.de/reputer/) to annotate repeat sequences in the plastomes of the 25 *Scrophularia* species (Kurtz and Schleiermacher, 1999). This analysis included four types of repeats: forward repeats, reverse repeats, palindromic repeats, and complementary repeats. The parameter settings were as follows: a Hamming distance of 3, a minimum repeat size of 30 bp, and a maximum repeat count of 80. For the analysis of simple sequence repeats (SSRs) in the *Scrophularia* plastomes, we employed MISA (https://pgrc.ipk-gatersleben.de/misa/) with the following parameter settings for the minimum repeat unit sizes: mononucleotide repeats of 10, dinucleotide repeats of 6, trinucleotide repeats of 4, tetranucleotide repeats of 3, pentanucleotide repeats of 3, and hexanucleotide repeats of 3 (Beier et al., 2017).

An analysis of relative synonymous codon usage (RSCU) and effective number of codon (ENC) was conducted using CodonW V1.4.2 (https://codonw.sourceforge.net/). RSCU was employed to assess variations in the usage patterns of synonymous codons across the entire genome. It reflects the ratio of the observed frequency of a particular synonymous codon in the actual gene sample to its expected average frequency based on theoretical calculations (Sharp and Li, 1987; Chen et al., 2022a). The cusp program from the EMBOSS (https://emboss.toulouse.inra.fr/) website was used to compute the GC content associated with the three positions of codons (first, second, and third) in the entire plastome and within 25 plastomes (Rice et al., 2000).

### 2.3 Comparative plastome analysis and contraction or expansion of inverted repeats

To elucidate the intergenic and intra-species variations and gene structural composition in 25 species of East Asian *Scrophularia*, with *S. alaschanica* as the reference sequence, we employed mVISTA (https://genome.lbl.gov/vista/mvista/submit.shtml/) for genome-wide multiple sequence alignment. We utilized the shuffle-LAGAN mode, which enables global alignment and is the only mode capable of identifying gene rearrangements and inversions (Brudno et al., 2003; Frazer et al., 2004). Additionally, we conducted collinearity analysis using Mauve for multi-genome alignment of the *Scrophularia* species, aiming to detect rearrangements and inversions (Darling et al., 2004).

Variation in the size of the molecule is typically due to the expansion or contraction of the inverted repeat (IR) into or out of adjacent single-copy regions, as well as changes in sequence complexity caused by insertions or deletions of unique sequences (Plunkett and Downie, 2000). CPJSdraw online software (https://cloud.genepioneer.com:9929/#/tool/alltool/detail/335) was used to compare IR border expansion or contraction of the twenty-five sequences by directly uploading their gb format files (Li et al., 2023a).

### 2.4 Selective pressure analysis and nucleotide diversity analysis

We used a Perl script to extract protein-coding genes from each *Scrophularia* plastome. These sequences were then visualized and examined for divisibility by three using Geneious software. Using *S. takesimensis* (KP718628) as the reference sequence, we calculated the Ka/Ks values for each CDS using TBtools V1.113 (Chen et al., 2020).

CDS and intergenic spacers (IGS) were extracted with a Perl script (https://github.com/quxiaojian/Bioinformatic_Scripts/tree/master/extract_sequences_from_gb_files) and organized using Geneious software. The CDS and IGS were separately aligned using MAFFT v7.0 to construct matrices (Katoh and Standley, 2013). Using *S. takesimensis* (KP718628) as the reference sequence, nucleotide diversity analysis was performed in DnaSP v6.0 to determine the total number of mutations (Eta) and nucleotide diversity (Pi) in the 25 *Scrophularia* plastomes (Rozas et al., 2017).

### 2.5 Phylogenetic analysis

A set of 44 plastomes from six genera in Scrophulariaceae, which included 34 individuals in *Scrophularia*, 2 in *Verbascum*, 2 in *Buddleja*, 2 in *Eremophila*, 2 in *Myoporum*, and 1 in *Leucophyllum*, as well as one in Plantaginaceae, were used. Aside from the *Scrophularia* species, the remaining 10 species are outgroups, with *Digitalis lanata* used as the root of the phylogenetic tree (**Supplementary Table 3**). These were chosen to construct two phylogenetic trees inferred from whole plastome data and from CDS data only, respectively. MAFFT V7 was used to align 44 plastome sequences under default parameters. IQ-TREE V1.6.8 was used to construct phylogenetic trees using the maximum likelihood method (Nguyen et al., 2015; Yang et al., 2022a). When running IQ-TREE, it will be executed twice: the first run is to select the best model, and the second run is to construct the tree using the best model. The preferred model for the whole genome was TVM+F+R2, while UNREST+FO+R2 was the optimal model for constructing phylogenetic trees using CDS. Based on Bayesian Information Criterion (BIC), the best models for both whole genome and CDS trees were confirmed as TVM+I+G substitution models using jModelTest v2.1.10 (Darriba et al., 2012). MrBayes V3.2.7 was employed for Bayesian inference phylogenetic tree construction (Ronquist and Huelsenbeck, 2003). The analyses were conducted with 2 million generations using the Markov Chain Monte Carlo (MCMC) algorithm. Trees were sampled every 100 iterations. The first 1/4 of the calculated trees were discarded as burn-in, and a consensus tree was constructed from the remaining trees to compute posterior probabilities (PPs).

## 3 Results

### 3.1 Sequencing, plastome structure and characteristics

**Figure 1:**
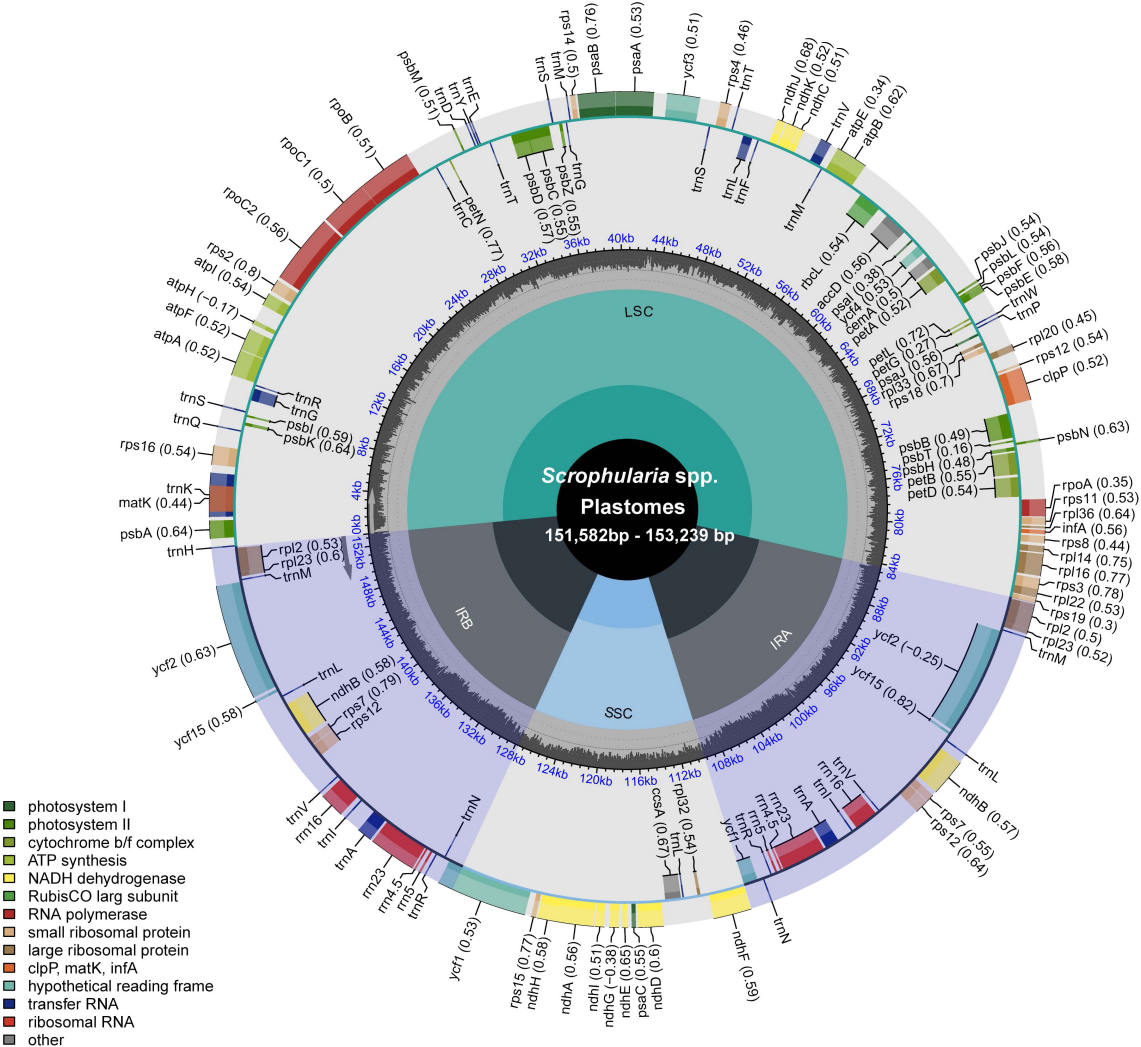
Circular gene map of *Scrophularia* plastomes. The map illustrates the characteristic quadruple structure of the plastome, where the blue regions represent the IR regions, and the gray regions represent the LSC and SSC regions. The transcription directions for the inner and outer genes are clockwise and anticlockwise, respectively. The black, uneven circle in the middle represents the GC content along the genome. The functional classification of the genes is shown in the left bottom corner.

The quality metrics of raw reads (Q20 and Q30) and clean reads (reads after quality trimming, reads assembled and coverage of assemblies) indicated good sequencing quality and high depth coverage, demonstrating that the sequencing depth was sufficient to support the assembly of the plastid genome (**Supplementary Table 4**). After assembly and annotation, we obtained the following structural information of the plastid genome. In the 25 East Asian *Scrophularia* species, plastomes had a total length ranging from 151,582bp to 153,239 bp. It consisted of a large single-copy region (LSC) spanning from 82,790 bp to 84,386 bp, a small single-copy region (SSC) ranging from 17,321 bp to 17,942 bp, and two IR regions with lengths between 25,392 bp and 25,570 bp. The *Scrophularia* plastomes encoded a total of 132 genes, comprising 18 duplicated genes and 114 unique genes, with 80 protein-coding genes, 4 ribosomal RNA (rRNA) genes and 30 transfer RNA (tRNA) genes. Among 114 unique genes, 10 protein-coding genes (*petB, petD*, *atpE*, *ndhB* (x2), *rpl16*, *rpl2* (x2), *rps16*, *rpoC1*) and 5 tRNA genes (*trnA-UGC*, *trnG-GCC*, *trnI-GAU, trnL-UAA*, *trnV-UAC*) contained a single intron, while 3 genes (*clpP*, *rps12*, and *ycf3*) had 2 introns (**Figure 1**; **Supplementary Table 5**). These characteristics align with previous findings in *Scrophularia* plastomes (Wang et al., 2022; Guo et al., 2023b). The overall GC content ranged from 37.87% to 38.09%. The GC content in the LSC ranged from 35.93% to 36.20%, while in the SSC it ranged from 32.04% to 32.35%. The IR regions exhibited a GC content ranging from 43.08% to 43.22%, which was higher than that of both the LSC and SSC regions (**Table 1**).

**Table 1:** Basic features of East Asian *Scrophularia* plastomes.

Species	GenBank Acc. No.	Total plastome size (bp)	LSC length	SSC length	IR length	Total GC content (%)	LSC	SSC	IR
*S. alaschanica*	OR393409	151,997	83,190	17,913	25,447	38.04	36.19	32.08	43.17
*S. amgunensis*	OR393399	153,173	84,331	17,890	25,476	37.95	36.04	32.14	43.16
*S. buergeriana*	OQ633013	153,148	84,259	17,925	25,482	37.98	36.07	32.17	43.18
*S. chasmophila*	OR393407	152,335	83,874	17,321	25,570	38.01	36.09	32.27	43.11
*S. delavayi*	OR393414	153,050	84,171	17,877	25,501	37.98	36.07	32.16	43.16
*S. elatior*	OR393401	153,239	84,386	17,905	25,474	37.96	36.04	32.21	43.16
*S. fargesii*	OR393413	152,429	83,577	17,900	25,476	38.07	36.18	32.26	43.21
*S. henryi*	OR393406	153,028	84,144	17,942	25,471	38.00	36.10	32.19	43.18
*S. heucheriiflora*	OR393400	152,536	83,660	17,888	25,494	37.97	36.11	32.13	43.08
*S. hypsophila*	OR393420	152,080	83,626	17,466	25,494	38.00	36.09	32.08	43.15
*S. jinii*	OR393405	152,313	83,469	17,892	25,476	38.09	36.20	32.35	43.19
*S. kakudensis*	OQ633012	153,032	84,138	17,922	25,486	37.98	36.08	32.15	43.17
*S. lijiangensis*	OR393402	152,668	83,965	17,919	25,392	38.01	36.11	32.17	43.22
*S. mandarinorum*	OR393419	152,879	84,151	17,918	25,405	37.98	36.07	32.18	43.19
*S. mapienensis*	OR393411	153,153	84,239	17,926	25,494	37.98	36.07	32.16	43.17
*S. modesta*	OR393403	152,997	84,134	17,903	25,480	38.00	36.09	32.16	43.18
*S. moellendorffii*	OR393418	151,582	82,790	17,906	25,443	38.03	36.16	32.06	43.18
*S. musashiensis*	OR393404	152,401	83,551	17,904	25,473	38.03	36.13	32.23	43.17
*S. ningpoensis*	OQ633009	153,173	84,255	17,938	25,490	37.99	36.08	32.18	43.19
*S. spicata*	OR393415	152,887	84,160	17,919	25,404	37.98	36.07	32.14	43.19
*S. stylosa*	OR393416	152,944	84,089	17,893	25,481	37.98	36.07	32.18	43.18
*S. taihangshanensis*	OR393412	153,221	84,373	17,936	25,456	37.87	35.93	32.04	43.13
*S. wattii*	OR393408	152,495	84,096	17,471	25,464	37.97	36.04	32.17	43.14
*S. yoshimurae*	OR393417	153,173	84,274	17,925	25,487	37.98	36.07	32.18	43.18
*S. yunnanensis*	OR393410	152,711	83,871	17,862	25,489	37.96	36.06	32.11	43.13

### 3.2 Repeat sequence, SSRs and codon usage bias analysis

**Figure 2:**
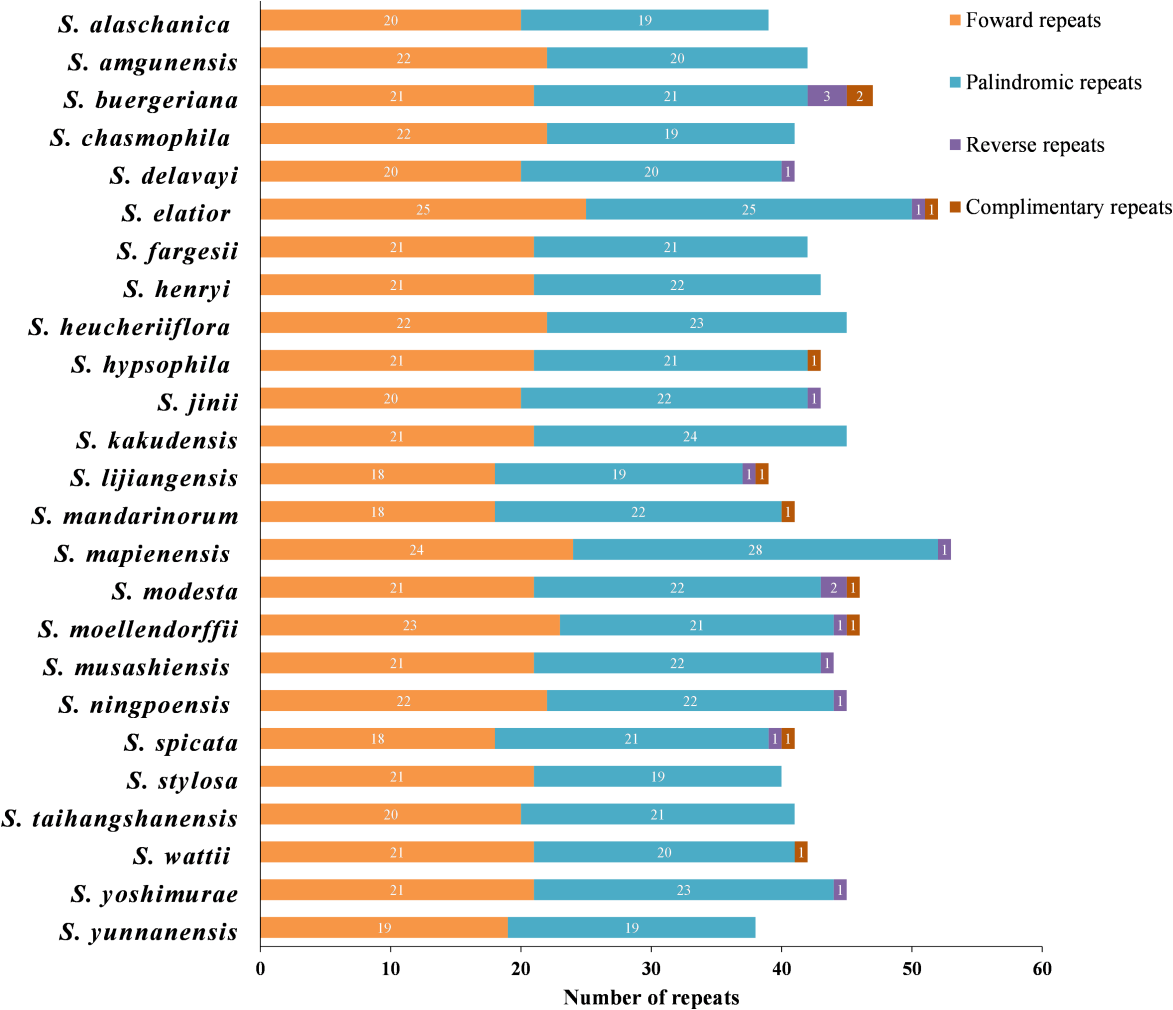
Quantitative analysis of four types of repeats in 25 East Asian *Scrophularia* plastomes.

A total of 1084 repeats were detected, consisting of 523 forward repeats, 536 palindromic repeats, 15 reverse repeats, and 10 complementary repeats (**Supplementary Table 6**). The lengths of the repeat sequences in the 25 *Scrophularia* plastomes varied from 38 (*S. yunnanensis*) to 53 (*S. mapienensis*). Among them, only 12 exhibited reverse repeats, while 9 had complementary repeats. *S. buergeriana* displayed the highest number of reverse repeats (3) and complementary repeats (2) (**Figure 2**). Regarding the base size, the majority of the dispersed repeat sequences were 30-39 bp in length, accounting for 79.61% of the total. A minority of repeats were 50 bp or longer (1.75%), with *S. lijiangensis*, *S. spicata*, and *S. mandarinorum* having the highest number (4) of dispersed repeats longer than 50 bp (**Supplementary Table 6**).

**Figure 3:**
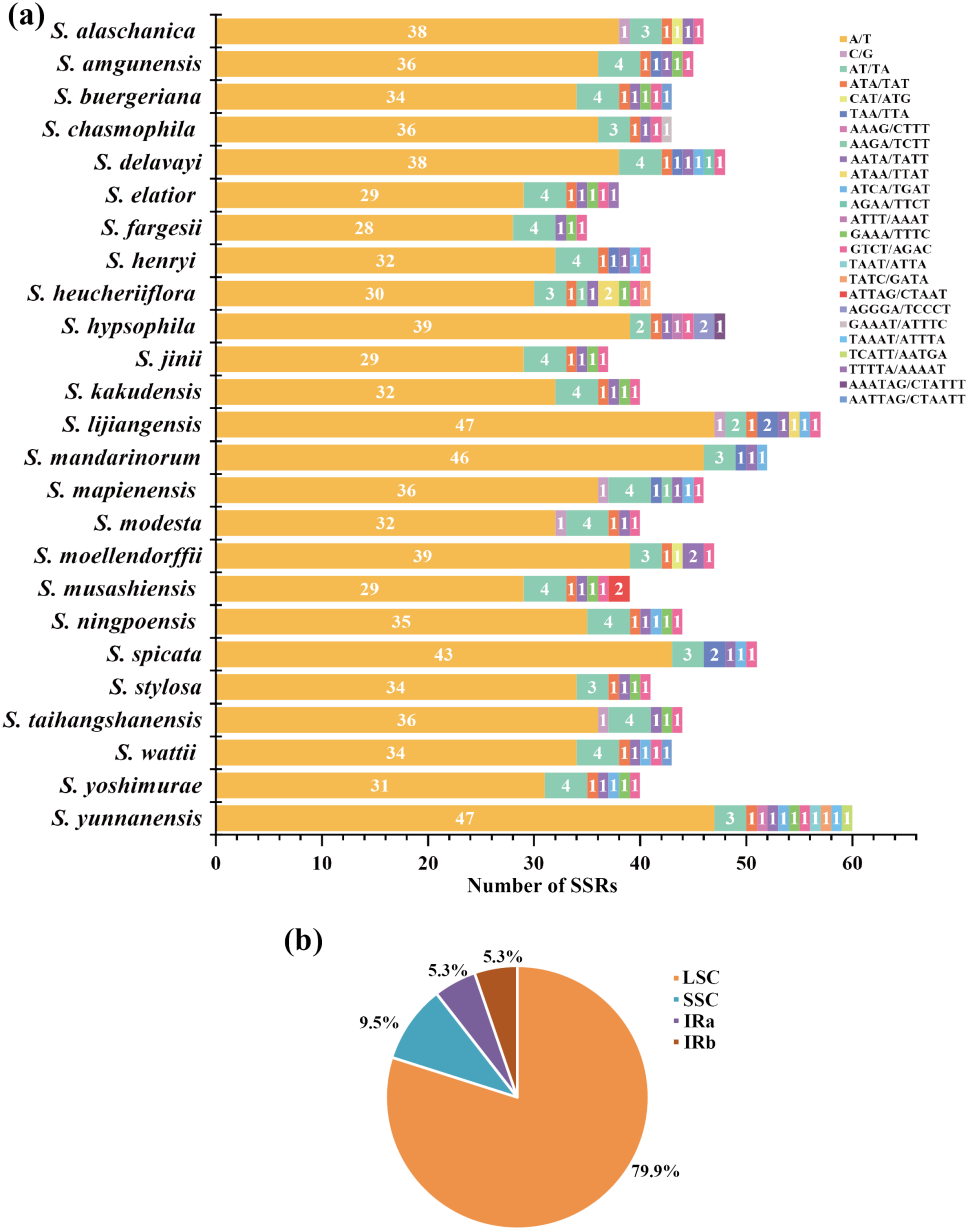
**(A)** Type and number of SSRs in 25 East Asian *Scrophularia* plastomes. **(B)** SSRs locus distribution (including LSC, SSC, IRa and IRb) of East Asian *Scrophularia* plastomes.

We observed a range of SSRs quantities in the plastomes of 25 East Asian *Scrophularia* species, with counts varying from 38 (*S. musashiensis* & *S. fargesii*) to 61 (*S. yunnanensis*). Among these SSRs, 1040 (93.86%) were comprised of A/T bp, the frequency of C and G nucleotides was low (0.45%). Besides single nucleotide repeats, we identified 10 different types of SSRs shared among the 25 *Scrophularia* plastomes, namely AT/TA, ATA, CAT, TTA, AAGA, AATA, ATCA, GAAA, and GTCT (**Figure 3A**, **Supplementary Table 7**). It is worth noting that the SSRs exhibited nucleotide variations, with each type of repeat (di-, tri-, tetra-, penta-, and hexanucleotide) corresponding to 7.9%, 2.8%, 7.6%, 0.7% and 0.2%, respectively (**Supplementary Table 7**). The SSRs were unevenly distributed in the plastome, with 79.9% located in the LSC region, 9.5% in the SSC region, and 10.6% in the IRb and IRa regions, indicating increased polymorphic variation in LSC region (**Figure 3B**).

**Figure 4:**
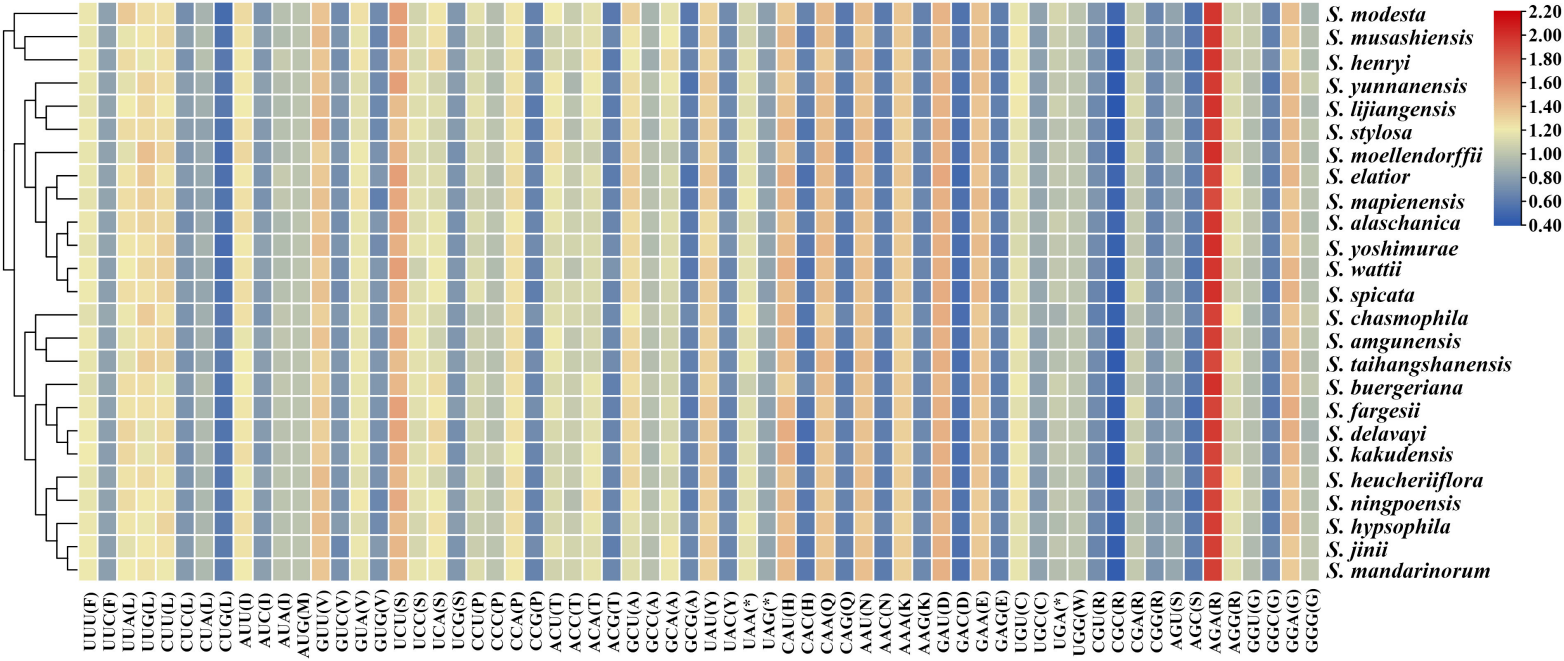
Heatmap of Relative Synonymous Codon Usage (RSCU) values for 25 East Asian *Scrophularia* plastomes.

We conducted a statistical analysis of relative synonymous codon usage (RSCU) in 25 East Asian *Scrophularia* species, and the results were consistent across different species. The most and least frequently used amino acids were Leucine (Leu) (126,428) and Tryptophan (Trp) (17,404), respectively. Among the 30 codons analyzed, the RSCU values for each *Scrophularia* individual were greater than 1, indicating a preference for these codons. Among these preferential codons, the codon for Arginine (AGA) exhibited the highest preference, with an average RSCU value of 1.95. The codons UGG and AUG did not show any preference (RSCU = 1) (**Figure 4**; **Supplementary Table 8**). The effective number of codons (ENC) value typically ranges from 20 to 61, with lower values indicating stronger bias in codon usage away from random selection (Wu et al., 2007; Li et al., 2023b). Our research results revealed an ENC range of 55.36 (*S. elatior*) to 56.19 (*S. hypsophila*) across the 25 *Scrophularia* plastomes, with an average of 55.78.

### 3.3 Comparative plastome analysis and contraction or expansion of inverted repeats

Through the whole-genome alignment, we found minimal differences between the intra-genic and intergenic regions across the global view (**Supplementary Figure 1**). To analyze this further, we used Mauve for collinearity analysis, where homologous regions were shown in the same color. The plastome sequences of the 25 *Scrophularia* species displayed normal and single blocks. The homologous blocks were aligned in a linear manner, indicating a lack of rearrangements and inversions, resulting in good collinearity (**Supplementary Figure 2**).

**Figure 5:**
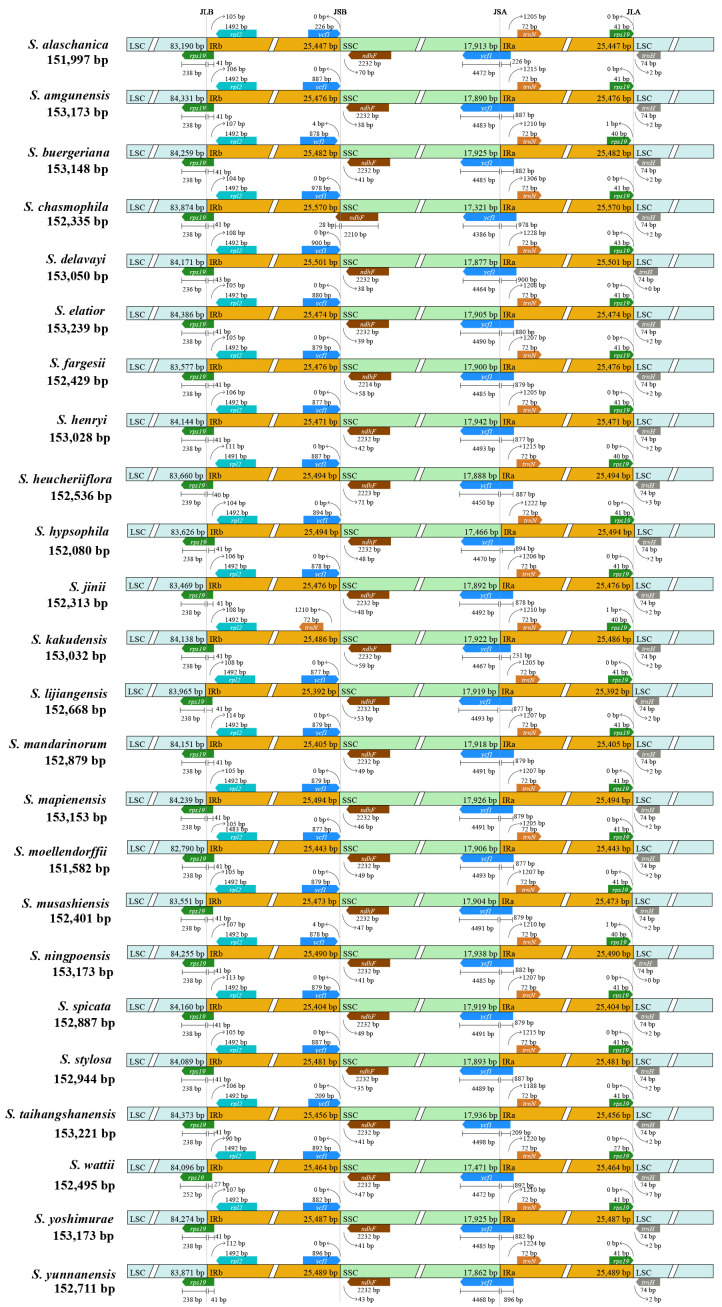
The contraction and expansion diagram of the IR region in the plastomes. The positions of LSC, IR, and SSC junctions were compared among 25 *Scrophularia* species. JLB stands for the junction between the long single copy and reverse repeat (LSC/IRb), JSB stands for the junction between reverse repeat and short single copy (IRb/SSC), JSA stands for the junction between short single copy and forward repeat (SSC/IRa), and JLA stands for the junction between forward repeat and long single copy (IRa/LSC).

Comparison of 25 plastomes of East Asian *Scrophularia* species revealed minor variations in the expansion and contraction of the IR regions. The IRa/SSC and IRb/LSC boundaries extended into the *ycf1* and *rps19* genes, leading to the generation of pseudogenes. In IRa, the length of the pseudogene *ycf1* ranged from 209 to 978 bp, with the majority being around 870 bp. Specifically, *S. chasmophila* had a pseudogene *ycf1* length of 978 bp, *S. delavayi* had 900 bp, and *S. kakudensis* had 231 bp. While the *ycf1* gene, in those species, had a length ranging from 4,386 to 4,493 bp in the SSC region. Except for *S. kakudensis*, *S. buergeriana*, and *S. ningpoensis*, the pseudogene *rps19* genes in the IRa region were at a distance of 0 bp from the LSC/IRa junction (JLA). The *rps19* gene located at the JLB extended into the LSC region with a length of 238-252 bp (252 bp in *S. wattii*). In *S. moellendorffii*, the *rpl2* gene completely situated in the IRb region experienced a slight contraction. It had a length of 1,483 bp, distinguishing it from the 1,492 bp length found in other species. Notably, in *S. chasmophila*, the *ndhF* gene expanded from the SSC region into the IRb region with an expansion length of 28 bp, while its length in the SSC region was 2,210 bp. In contrast, in the remaining species, the *ndhF* genes were entirely within the SSC region, with lengths mostly around 2,232 bp (**Figure 5**).

### 3.4 Selective pressure analysis and nucleotide diversity analysis

**Figure 6:**
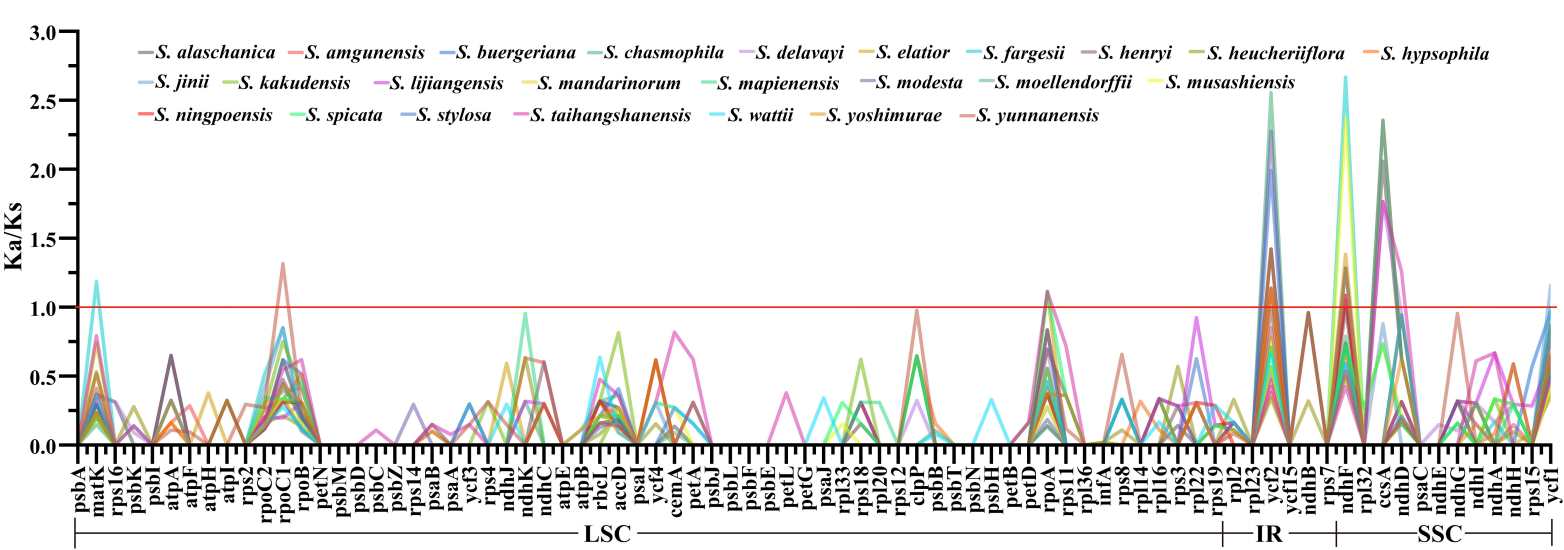
Ka/Ks Analysis of 80 CDS in 25 East Asian *Scrophularia* Species.

A selection pressure analysis was performed on the protein-coding genes of 25 East Asian *Scrophularia* plastomes. Among the 80 analyzed protein-coding genes, the average Ka/Ks ratio was found to be 0.1172. The most conserved genes showed an average Ka/Ks value of 0 (excluding NA, where NA indicates Ks = 0, meaning no synonymous mutations), indicating strong purifying selection pressure. These genes include *petB*, *petG*, *petN*, *psaC*, *psbA*, *psbD*, *psbE*, *psbI*, *psbJ*, *psbL*, *psbM*, *psbN*, *psbT*, *psbZ*, and *rps7*. The top three Ka/Ks values were 2.6677 for *ndhF* gene in *S. chasmophila*, 2.5567 for *ycf2* gene in *S. fargesii*, and 2.3657 for *ndhF* gene in *S. musashiensis* (**Figure 6**; **Supplementary Table 9**).

**Figure 7:**
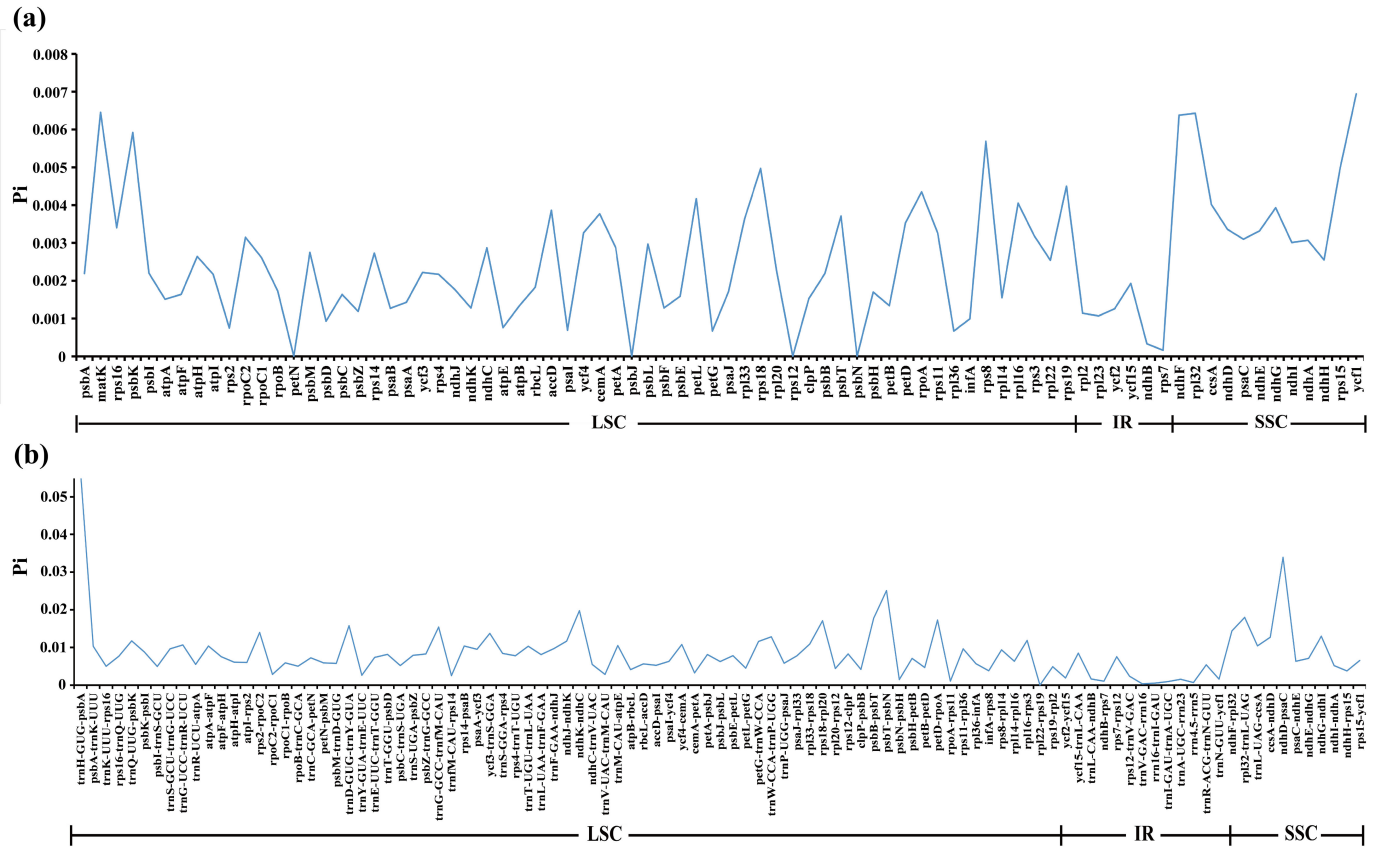
Comparison of nucleotide diversity (Pi) values. **(A)** among 80 CDS of 25 East Asian *Scrophularia* species. **(B)** among 101 IGS of 25 East Asian *Scrophularia* species.

Using a Perl script, we extracted 80 CDS and 101 IGS from the plastomes of 25 East Asian *Scrophularia* species. Subsequently, nucleotide diversity (Pi) analysis was conducted using DnaSP. The Pi values ranged from 0 to 0.00694 in CDS and from 0 to 0.005691 in IGS. The IGS exhibited higher levels of polymorphism compared to the CDS. Higher Pi values were observed in CDS such as *ycf1* (0.00694), *matK* (0.00645), *rpl32* (0.00643), *ndhF* (0.00638), *psbK* (0.00592), and *rps8* (0.00569) (**Figure 7A**; **Supplementary Table 10**). Similarly, IGS including *trnH-GUG*-*psbA* (0.05691), *ndhD*-*psaC* (0.03392), *psbT*-*psbN* (0.02508), *ndhK*-*ndhC* (0.01976), *rpl32*-*trnL-UAG* (0.01798), *psbB*-*psbT* (0.01778), *petD*-*rpoA* (0.01726), and *rps18*-*rpl20* (0.01709) exhibited higher Pi values (**Figure 7B**; **Supplementary Table 10**).

### 3.5 Phylogenetic analysis

**Figure 8:**
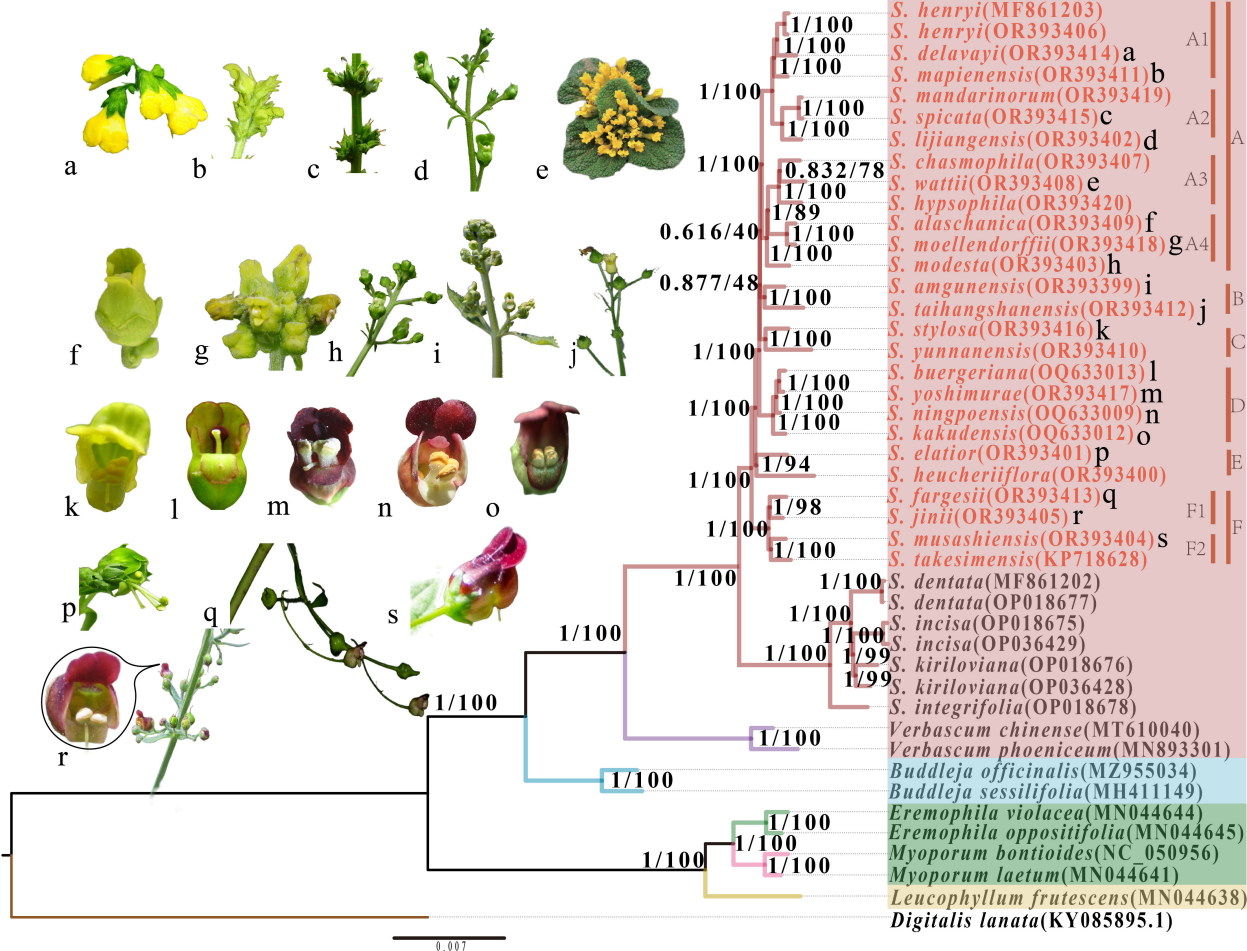
A phylogenetic tree of 30 *Scrophularia* species inferred from maximum likelihood based on the plastome sequence dataset. Support values above the branches, assessed by two methods (ML, BI), are listed as the order PP (posterior probability)/BS (bootstrap support). Genbank accession numbers of all species are given within the parentheses. Rectangular blocks of red, blue, green, and yellow represent the tribes Scrophularieae, Buddlejeae, Myoporeae and Leucophylleae, respectively. The four tribes all belong to the Scrophulariaceae, while *Digitalis lanata* belongs to the Plantaginaceae. The red branches represent *Scrophularia*, with the remaining species being outgroups. Within the red block, red font indicates *Scrophularia* sect. *Scrophularia*, while black font indicates *Scrophularia* sect. *Caninae*. The capital letters **A-F** represent different clades within *S.* sect. *Scrophularia* studied. Lower case letters **a-s** correspond to images of some species given on the left.

Based on the analysis of complete plastome sequences and CDS data from 44 species, we constructed four phylogenetic trees using Bayesian inference (BI) and maximum likelihood (ML) methods. The topologies of these trees were so similar that we decided to show the tree inferred from the Maximum Likelihood method (**Figure 8**; **Supplementary Figure 3**). The majority of nodes received strong support (BI-PP/ML-BS = 1/100). Scrophulariaceae species throughout the tree could be categorized into three distinct monophyletic groups, including tribe Scrophularieae, tribe Buddlejeae, and tribes Myoporeae + Leucophylleae. The genus *Scrophularia* could be further divided into two sections: *Scrophularia* sect. *Caninae* and *S.* sect. *Scrophularia*; the 25 newly studied species belong to the latter section. The phylogenetic tree robustly supported that the 25 species fell into six monophyletic clades (A-F). Clade F was sister group to clades A-E. Within the core of the phylogenetic tree, the other clades formed a topology of [clade C + (clade A + B)].

## 4 Discussion

The plastome structure is generally conserved. This conservation indicates the presence of widespread evolutionary selective pressures associated with photosynthesis, which is the primary function of chloroplasts (Bungard, 2004). The majority of nonsynonymous substitutions are under purifying selection, implying that in most cases, natural selection eliminates harmful mutations and maintains amino acids unchanged (Hughes et al., 2008). In our study, the Ka/Ks values of most of genes were less than 1 (98.45%) in these *Scrophularia* species, while a few genes were greater than 1 (1.55%). It suggests that the majority of genes in the *Scrophularia* plastome are under purifying selection, with a few under significant positive selection, such as *ycf2* gene (*S. modesta, S. chasmophila, S. alaschanica, S. stylosa, S. yoshimurae, S. moellendorffii, S. hypsophila, S. buergeriana, S. ningpoensis*) and *ndhF* gene (*S. elatior, S. musashiensis, S. chasmophila, S. fargesii, S. delavayi, S. stylosa, S. yoshimurae, S. buergeriana, S. ningpoensis*) (**Figure 6**).

Repetitive plastome sequences are crucial for genome rearrangement and genetic variation (Qi et al., 2017; Wei et al., 2020; Yang et al., 2022b). Within our study, a total of 1,084 repeat sequences were detected, the loci containing these repetitive sequences are critical hotspots for genomic reconfiguration. They provide information for understanding the evolutionary history and sequence divergence of plant species (Zong et al., 2019; Sun et al., 2020; Chong et al., 2022). Furthermore, they may provide abundant information for population studies and phylogenetic analysis (Gao et al., 2009; Nie et al., 2012; Zong et al., 2019). Of SSRs, 1040 (93.86%) consisted of A/T bp, with a notably low frequency of C and G nucleotides (0.45%), a pattern also observed in other medicinal plants such as *Gentiana* (Ni et al., 2017), *Alpinia* (Li et al., 2020), and *Aconitum* (Niu et al., 2023). SSRs in the plastome are frequently used as genetic markers in population genetics and evolutionary studies (Yang et al., 2016; Guo et al., 2020; Chen et al., 2023). Among the 25 samples of *Scrophularia*, a total of 7 common SSRs loci were identified. Analyzing similar SSRs in comparable genetic regions could be a potential approach for marking East Asian *Scrophularia* species for future population genetics studies, germplasm evaluation and resource conservation.

DNA sequence data from diverse organisms clearly show that synonymous codons for any given amino acid are not used with equal frequency, even though the choices among codons should be equivalent in terms of protein structure (Ikemura, 1985). The genomic GC content emerged as the strongest single determinant of codon usage variation across species (Plotkin and Kudla, 2011). The average GC content of the first, second, and third codon positions in the plastomes was 37.93%, 37.89%, and 38.15%, respectively. The overall GC3 content ranged from 37.87% (*S. taihangshanensis*) to 38.09% (*S. jinii*), all below 50%. There are a total of 28 codons with RSCU values greater than 1, of which 25 end with A/U, indicating a preference for codons ending in A/U in the plastomes of the 25 East Asian *Scrophularia* species. (**Supplementary Table 8**). The result is consistent with our previous research on the plastomes of *Scrophularia* (Wang et al., 2022; Guo et al., 2023b). Although the number and order of genes in the plastome are generally conserved, the IR regions expansion and contraction, a phenomenon known as “ebb and flow in plastomes”, is the main mechanism responsible for the variation in the plastome length of higher plant (Goulding et al., 1996; Kim and Lee, 2004; Zhu et al., 2016; Yin et al., 2018). Similar to many medicinal plants, the high GC content in the IR region may be caused by the elevated GC content of rRNA and tRNA in this region (Deng et al., 2021; Lu et al., 2022), as observed in various species such as *Salvia* (Liang et al., 2019), *Polygonum* (Guo et al., 2022), and *Atractylodes* (Xu et al., 2023). Additionally, the GC content in the IR regions is higher than in the LSC and SSC regions. Moreover, among 14 regions with higher Pi values, the majority were located in the LSC (64.28%), while a smaller portion was found in the SSC (35.72%). Notably, none of them were found in the IR regions. Overall, the IR displayed a lower level of polymorphism. This is consistent with previous reports, indicating that the IR regions are more conserved compared to the LSC and SSC regions (Wang et al., 2022; Guo et al., 2023b).

Comparative genomic analysis can contribute to gaining a comprehensive understanding of a genus (Sivashankari and Shanmughavel, 2007; Tonti-Filippini et al., 2017). Compared to protein-coding regions, the non-coding regions exhibited higher diversity and variability. The regions with overall significant differences, which are *trnH-GUG*-*psbA*, *rps16*-*trnQ-UUG*, and *trnT-UGU*-*trnL-UAA*, are in good agreement with the Pi calculation results. Research has shown that polymorphic indels and nucleotides of *trnH-psbA* could be used to authenticate most *Kaempferia* species (Techaprasan et al., 2010). The *trnH-GUG*-*psbA* has been supported by studies as a DNA barcode sequence for distinguishing the medicinal plant *Datura metel* and its adulterants (Bi et al., 2022). Therefore, the divergent hotspot regions could serve as candidate markers for future identification analyses within the *Scrophularia* genus. Developing universal primers targeting these hotspot regions would be of importance in revealing the molecular phylogenetics and conservation genetics of other *Scrophularia* species (Jia et al., 2017; Kong et al., 2017; Liu et al., 2021).

According to phylogenetic tree, tribe Scrophularieae was sister to tribe Buddlejeae, and together they formed a sister group to tribes Myoporeae + Leucophylleae. The interspecies relationships among these tribes were reasonably resolved and have also been confirmed in previous literature (Wang, 2015; Gao, 2021). We subdivided *Scrophularia* sect. *Scrophularia* into six clades (A-F) and discussed them based on morphological characteristics in the following. Subclades A1 and A2 formed a clade. In subclade A1, the stamens of *S. mapienensis* are about the same length as the lower lip of the corolla, which is yellow-white or purple (**Figure 8b**). The stamens of the other two species are half the length of the lower lip, and their bell-shaped or spherical corollas are yellow-green or yellow (**Figure 8a**). In subclade A2, *S. spicata* and *S. mandarinorum*, as sister taxa, can grow to over 1 meter in height, and the corolla color of species within subclade A2 is green or yellow-green (**Figures 8c, d**). Subclades A3 (*S. chasmophila*, *S. wattii*, and *S. hypsophila*) and A4 (*S. alaschanica* and *S. moellendorffii*) were sister groups and together they formed a sister relationship with *S. modesta*, which has green or yellowish-green corolla (**Figure 8h**). With the exception of *S. chasmophila*, which features a yellow-green corolla, the species belonging to subclades A3 and A4 are characterized by their bright yellow corollas (**Figures 8e-g**). The species of *Scrophularia* within subclade A3 are all perennial small herbaceous plants, especially *S. wattii* with scalelike and rosette leaves appressed to ground (**Figure 8e**). Clade B included *S. taihangshanensis* and *S. amgunensis*, both of which have yellow-green corollas (**Figures 8i, j**). *S. stylosa* with light yellow corolla and obcordate staminodes (**Figure 8k**) was sister to *S. yunnanensis* with green corolla, the two species constituting clade C. *S. ningpoensis* and its common medicinal substitutes or adulterants of *S. buergeriana*, *S. kakudensis*, and *S. yoshimurae* formed a monophyletic clade D. The corolla of *S. buergeriana* is green and *S. kakudensis* has outside green and inside purplish brown corolla, while the corollas of the other two species are purple (**Figures 8l-o**). Clade E included *S. elatior* and *S. heucheriiflora*, with their stamens extending beyond the green corolla (**Figure 8p**). The endemic Chinese species *S. fargesii* and *S. jinii* are sister taxa (subclade F1), while the Japanese endemics *S. musashiensis* and Korean endemics *S. takesimensis* clustered as subclade F2. These two subclades shared a common ancestor, with purple-red corollas and slender flower stalks (**Figures 8q-s**). The cyme inflorescence of *S. fargesii* consists of 1-3 or 5 flowers, while that of *S. jinii* comprises 1-7 flowers. As for leaf margin, *S. jinii* is deeply double serrate but *S. fargesii* is unequally double serrate (Wang et al., 2018).

However, there were also branches with relatively low support rate and short length in the phylogenetic trees, such as the relationships between Clade C and Clade (A+B), as well as between Clade B and Clade A. We speculate that there may have been a rapid radiation. Given the potential for minimal genetic disparities among species during rapid differentiation, the swift evolutionary radiation of species in a condensed timeframe may contribute to diminished support rates within the phylogenetic tree (Guo et al., 2023a; Liu et al., 2024). Consequently, these branches with low supports may stem from the accumulation of multiple lineages over a short period. To validate this phenomenon, additional studies will be needed increasing the number of individuals and delving into the population genomics of the East Asian *Scrophularia*. By examining variation information, we can discuss the genetic structure, gene flow, speciation mechanisms, and evolutionary dynamics of East Asian *Scrophularia* populations.

It is also important to consider that, while plastid genomes are helpful for constructing phylogenetic trees, they still have certain limitations. For example, they do not adequately address issues such as ILS, hybridization, and whole genome duplication (McLay et al., 2023; Brown et al., 2024; Session, 2024; Wang et al., 2024). As we know, introgression and ILS are common mechanisms that lead to cytonuclear discordance. Although cytonuclear discordance is widespread and often considered an obstacle in phylogenetic and taxonomic studies, it can also provide valuable information (Duan et al., 2023). Currently, *S. ningpoensis* is the primary species cultivated on a large scale. Through genomic research on the East Asian *Scrophularia*, we anticipate gaining insights into the genus, which will aid in molecular breeding and unlock the medicinal potential of a broader range of *Scrophularia* species. It will contribute to the rational exploration, full utilization, and sustainable cultivation and harvesting of medicinal plant resources, ensuring their conservation and sustainable use for future generations.

## Data Availability

The datasets presented in this study can be found in online repositories. The names of the repository/repositories and accession number(s) can be found in the article/**Supplementary Material**.
