# Corrigendum: Comparative plastome analyses and evolutionary relationships of 25 East Asian species within the medicinal plant genus *Scrophularia* (Scrophulariaceae)

**DOI:** 10.3389/fpls.2025.1551071

**Published:** 2025-01-27

**Authors:** Xia Wang, Lei Guo, Lulu Ding, Leopoldo Medina, Ruihong Wang, Pan Li

**Affiliations:** ^1^ Zhejiang Province Key Laboratory of Plant Secondary Metabolism and Regulation, College of Life Sciences and Medicine, Zhejiang Sci-Tech University, Hangzhou, China; ^2^ Real Jardín Botánico, Consejo Superior de Investigaciones Científicas (CSIC), Madrid, Spain; ^3^ Laboratory of Systematic and Evolutionary Botany and Biodiversity, College of Life Sciences, Zhejiang University, Hangzhou, China

**Keywords:** *Scrophularia*, medicinal plant, East Asia, chloroplast genome, comparative analysis, phylogenomics

In the published article, there was an error in the **Funding** statement. The reference number for the Zhejiang Province Basic Public Welfare Research Program Project was incorrectly given as “LGH22H280005”. The correct Funding statement appears below.

“The author(s) declare financial support was received for the research, authorship, and/or publication of this article. This work was financially supported by the Zhejiang Province Basic Public Welfare Research Program Project (LGN22H280005), Key Research and Development Program of Zhejiang Province (grant no. 2023C02017), the Fundamental Research Funds of Zhejiang Sci-Tech University (24042128-Y), General Scientific Research Projects of the Department of Education of Zhejiang Province (No. Y202353132).”

The authors apologize for this error and state that this does not change the scientific conclusions of the article in any way. The original article has been updated.

