# Supplementary material for: Comparative plastome analyses and evolutionary relationships of 25 East Asian species within the medicinal plant genus Scrophularia (Scrophulariaceae)
Source: Front Plant Sci. 2024 Sep 3;15:1439206. doi: 10.3389/fpls.2024.1439206 (PMC11411265; doi:10.3389/fpls.2024.1439206)
Supplement: Supplementary file 1 [file Table1.docx]

Supplementary Table 1 Main distribution and regional characteristics of 25 species of *Scrophularia* in China.

| Species | Distribution | Regional Characteristics |
| --- | --- | --- |
| *S. alaschanica* | Inner Mongolia: Alxa, Alxa Left Banner; Qinghai: Xinghai; Ningxia: Yinchuan, Helan | Northwest China |
| *S. amgunensis* | Inner Mongolia: Zalantun Banner | North China |
| *S. buergeriana* | Beijing, Tianjin, Hebei, Inner Mongolia, Liaoning, Jilin, Heilongjiang, Jiangsu, Zhejiang, Anhui, Fujian, Jiangxi, Shandong, Henan, Hubei, Hunan, Guangxi, Shaanxi | North China, Northeast China, East China, Central China, South China |
| *S. chasmophila* | Sichuan: Tianquan, Dege, Baiyu, Xiangcheng; Yunnan: Lijiang, Lushui, Deqin; Tibet: Suoxian | Southwest China |
| *S. delavayi* | Sichuan, Yunnan | Southwest China |
| *S. elatior* | Sichuan, Guizhou, Yunnan, Tibet | Southwest China |
| *S. fargesii* | Jiangxi, Hubei, Chongqing, Sichuan, Shaanxi, Gansu | Central China, North China, Southwest China, Northwest China |
| *S. henryi* | Hubei, Sichuan | Central China, Southwest China |
| *S. heucheriiflora* | Xinjiang | Northwest China |
| *S. hypsophila* | Yunnan | Southwest China |
| *S. jinii* | Hubei | Central China |
| *S. kakudensis* | Liaoning, Jiangsu, Guangdong, Shaanxi, Gansu | Northeast China, East China, South China, Northwest China |
| *S. lijiangensis* | Yunnan | Southwest China |
| *S. mandarinorum* | Sichuan, Yunnan, Tibet | Southwest China |
| *S. mapienensis* | Sichuan | Southwest China |
| *S. modesta* | Beijing, Hebei, Shanxi, Heilongjiang, Henan, Shaanxi | North China, Northeast China, Central China |
| *S. moellendorffii* | Beijing, Hebei, Henan, Hubei, Sichuan | North China, Central China, Southwest China |
| *S. musashiensis* | Japan | — |
| *S. ningpoensis* | Beijing, Tianjin, Hebei, Shanxi, Liaoning, Heilongjiang, Jiangsu, Zhejiang, Anhui, Fujian, Jiangxi, Henan, Hubei, Hunan, Guangdong, Guangxi, Hainan, Chongqing, Sichuan, Guizhou, Yunnan, Shaanxi, Gansu, Xinjiang | North China, Northeast China, East China, Central China, South China, Southwest China, Northwest China |
| *S. spicata* | Sichuan, Yunnan, Tibet | Southwest China |
| *S. stylosa* | Shaanxi, Gansu | Northwest China |
| *S. taihangshanensis* | Henan | Central China |
| *S. wattii* | Tibet | Southwest China |
| *S. yoshimurae* | Taiwan | East China |
| *S. yunnanensis* | Sichuan, Yunnan | Southwest China |
