# Supplementary material for: Comparative plastome analyses and evolutionary relationships of 25 East Asian species within the medicinal plant genus Scrophularia (Scrophulariaceae)
Source: Front Plant Sci. 2024 Sep 3;15:1439206. doi: 10.3389/fpls.2024.1439206 (PMC11411265; doi:10.3389/fpls.2024.1439206)
Supplement: Supplementary file 3 [file Table3.docx]

Supplementary Table 3. The list of 44 plastomes in phylogenetic analyses.

| Family | Genus | Taxon | GenBank Accession |
| --- | --- | --- | --- |
| Scrophulariaceae | *Scrophularia* | *S. alaschanica* | OR393409 |
|  |  | *S. amgunensis* | OR393399 |
|  |  | *S. buergeriana* | OQ633013 |
|  |  | *S. chasmophila* | OR393407 |
|  |  | *S. delavayi* | OR393414 |
|  |  | *S. dentata* | MF861202 |
|  |  | *S. dentata* | OP018677 |
|  |  | *S. elatior* | OR393401 |
|  |  | *S. fargesii* | OR393413 |
|  |  | *S. henryi* | OR393406 |
|  |  | *S. henryi* | MF861203 |
|  |  | *S. heucheriiflora* | OR393400 |
|  |  | *S. hypsophila* | OR393420 |
|  |  | *S. incisa* | OP036429 |
|  |  | *S. incisa* | OP018675 |
|  |  | *S. integrifolia* | OP018678 |
|  |  | *S. jinii* | OR393405 |
|  |  | *S. kakudensis* | OQ633012 |
|  |  | *S. kiriloviana* | OP018676 |
|  |  | *S. kiriloviana* | OP036428 |
|  |  | *S. lijiangensis* | OR393402 |
|  |  | *S. mandarinorum* | OR393419 |
|  |  | *S. mapienensis* | OR393411 |
|  |  | *S. modesta* | OR393403 |
|  |  | *S. moellendorffii* | OR393418 |
|  |  | *S. musashiensis* | OR393404 |
|  |  | *S. ningpoensis* | OQ633009 |
|  |  | *S. spicata* | OR393415 |
|  |  | *S. stylosa* | OR393416 |
|  |  | *S. taihangshanensis* | OR393412 |
|  |  | *S. takesimensis* | KP718628 |
|  |  | *S. wattii* | OR393408 |
|  |  | *S. yoshimurae* | OR393417 |
|  |  | *S. yunnanensis* | OR393410 |
|  | *Verbascum* | *V. chinense* | MT610040 |
|  |  | *V. phoeniceum* | MN893301 |
|  | *Buddleja* | *B. officinalis* | MZ955034 |
|  |  | *B. sessilisfolia* | MH411149 |
|  | *Eremophila* | *E. oppositifolia* | MN044645 |
|  |  | *E. violacea* | MN044644 |
|  | *Myoporum* | *M. bontioides* | NC_050956 |
|  |  | *M. laetum* | MN044641 |
|  | *Leucophyllum* | *L. frutescens* | MN044638 |
| Plantaginaceae | *Digitalis* | *D. lanata* | KY085895.1 |
