# Supplementary material for: Comparative plastome analyses and evolutionary relationships of 25 East Asian species within the medicinal plant genus Scrophularia (Scrophulariaceae)
Source: Front Plant Sci. 2024 Sep 3;15:1439206. doi: 10.3389/fpls.2024.1439206 (PMC11411265; doi:10.3389/fpls.2024.1439206)
Supplement: Supplementary file 5 [file Table5.docx]

Supplementary Table 5. Gene composition of *Scrophularia* plastomes.

| **Groups of Gene** | **Name of Gene** |
| --- | --- |
| Ribosomal RNAs | *rrn16(×2), rrn23(×2), rrn23(×2), rrn5(×2)* |
| Transfer RNAs | *^a^ trnA-UGC(×2), trnC-GCA, trnD-GUC, trnE-UUC, trnF-GAA, trnfM-CAU*  *^a^ trnG-GCC, trnG-UCC, trnI-CAU(×2), trnH-GUG, ^a^ trnI-GAU(×2), trnK-UUU, trnL-CAA(×2)* |
|  | *^a^ trnL-UAA, trnL-UAG, trnM-CAU, trnN-GUU(×2), trnP-UGG, trnQ-UUG, trnR-ACG(×2)*  *trnR-UCU, trnS-GCU, trnS-GGA, trnS-UGA, trnT-GGU, trnT-UGU, trnV-GAC(×2),*  *^a^ trnV-UAC, trnW-CCA, trnY-GUA* |
| Photosystem I | *psaA, psaB, psaC, psaI, psaJ* |
| Photosystem II | *psbA, psbB, psbC, psbD, psbE, psbF, psbH, psbI, psbJ, psbK, psbL, psbM, psbN, psbT, psbZ* |
| Cytochrome | *petA, ^a^ petB, ^a^ petD, petG, petL, petN* |
| ATP synthase | *atpA, atpB, atpE, ^a^ atpF, atpH, atpI* |
| Rubisco | *rbcL* |
| NADH dehydrogenase | *ndhA, ^a^ ndhB(×2), ndhC, ndhD, ndhE, ndhF, ndhG, ndhH, ndhI, ndhJ, ndhK* |
| ATP-dependent protease subunit P | *^b^ clpP* |
| Chloroplast translational initiation factor | *infA* |
| Chloroplast envelope membrane protein | *cemA* |
| Large units | *rpl33, rpl20, rpl36, rpl14, ^a^ rpl16, ^a^ rpl2(×2), rpl23(×2), rpl32, rpl22* |
| Small units | *^a^ rps16, rps2, rps14, rps4, rps18, ^b^ rps12(×2), rps11, rps8, rps19, rps3, rps7(×2), rps15* |
| RNA polymerase | *rpoC2, ^a^ rpoC1, rpoB, rpoA* |
| Miscellaneous proteins | *matK, accD, ccsA* |
| Hypothetical proteins and conserved reading frame | *^b^ ycf3, ycf4, ycf2(×2), ycf1, ycf15(×2)* |

a Indicates the genes containing a single intron. b Indicates the genes containing two introns. (×2) Indicates genes duplicated in the IR regions.
