# Supplementary figures and images for: Comparative plastome analyses and evolutionary relationships of 25 East Asian species within the medicinal plant genus Scrophularia (Scrophulariaceae)
Source: Front Plant Sci. 2024 Sep 3;15:1439206. doi: 10.3389/fpls.2024.1439206 (PMC11411265; doi:10.3389/fpls.2024.1439206)

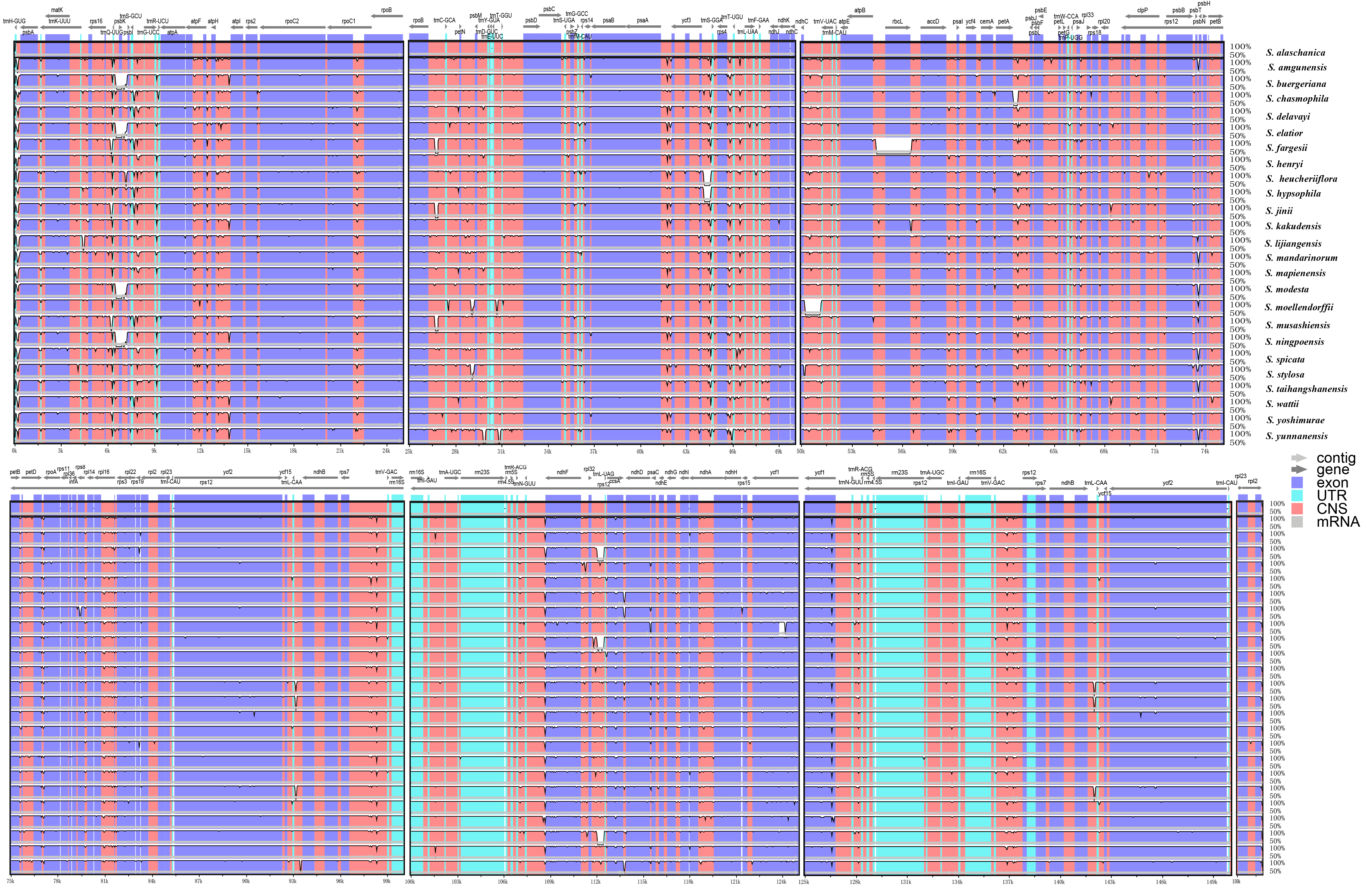

Supplement: Supplementary file 11 [file Image1.jpeg]

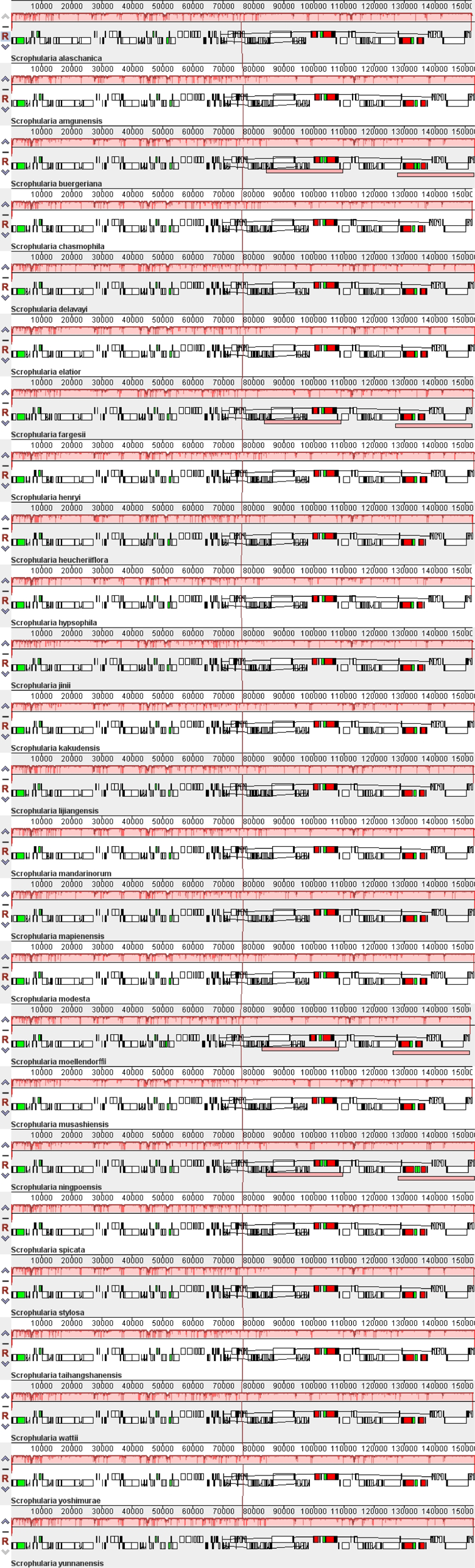

Supplement: Supplementary file 12 [file Image2.jpeg]

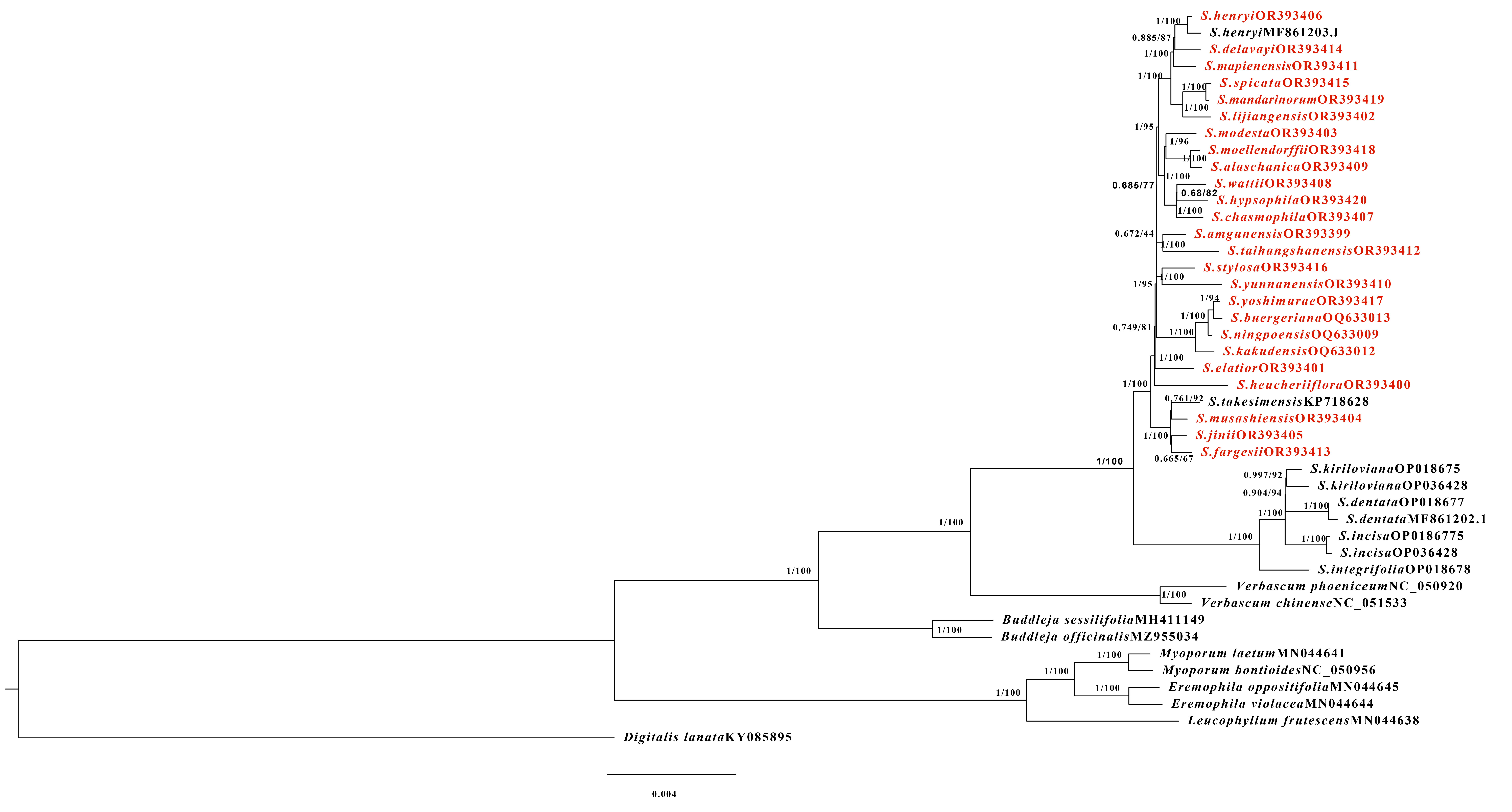

Supplement: Supplementary file 13 [file Image3.jpeg]
